# Stability selection enhances feature selection and enables accurate prediction of gestational age using only five DNA methylation sites

**DOI:** 10.1186/s13148-023-01528-3

**Published:** 2023-07-13

**Authors:** Kristine L. Haftorn, Julia Romanowska, Yunsung Lee, Christian M. Page, Per M. Magnus, Siri E. Håberg, Jon Bohlin, Astanand Jugessur, William R. P. Denault

**Affiliations:** 1 Centre for Fertility and Health, Norwegian Institute of Public Health, Oslo, Norway; 2 Institute of Health and Society, University of Oslo, Oslo, Norway; 3 Department of Global Public Health and Primary Care, University of Bergen, 5020 Bergen, Norway; 4 Division for Mental and Physical Health, Department of Physical Health and Aging, Norwegian Institute of Public Health, Oslo, Norway; 5 Division for Infection Control and Environmental Health, Department of Infectious Disease Epidemiology and Modelling, Norwegian Institute of Public Health, Oslo, Norway; 6 Department of Human Genetics, University of Chicago, Chicago, IL 60637 USA

**Keywords:** DNA methylation, Epigenetics, Gestational age, Illumina MethylationEPIC BeadChip, Epigenetic clock, Stability selection, Feature selection, MoBa, MBRN, Cord blood

## Abstract

**Background:**

DNA methylation (DNAm) is robustly associated with chronological age in children and adults, and gestational age (GA) in newborns. This property has enabled the development of several epigenetic clocks that can accurately predict chronological age and GA. However, the lack of overlap in predictive CpGs across different epigenetic clocks remains elusive. Our main aim was therefore to identify and characterize CpGs that are stably predictive of GA.

**Results:**

We applied a statistical approach called ‘stability selection’ to DNAm data from 2138 newborns in the Norwegian Mother, Father, and Child Cohort study. Stability selection combines subsampling with variable selection to restrict the number of false discoveries in the set of selected variables. Twenty-four CpGs were identified as being stably predictive of GA. Intriguingly, only up to 10% of the CpGs in previous GA clocks were found to be stably selected. Based on these results, we used generalized additive model regression to develop a new GA clock consisting of only five CpGs, which showed a similar predictive performance as previous GA clocks (*R*^2^ = 0.674, median absolute deviation = 4.4 days). These CpGs were in or near genes and regulatory regions involved in immune responses, metabolism, and developmental processes. Furthermore, accounting for nonlinear associations improved prediction performance in preterm newborns.

**Conclusion:**

We present a methodological framework for feature selection that is broadly applicable to any trait that can be predicted from DNAm data. We demonstrate its utility by identifying CpGs that are highly predictive of GA and present a new and highly performant GA clock based on only five CpGs that is more amenable to a clinical setting.

**Supplementary Information:**

The online version contains supplementary material available at 10.1186/s13148-023-01528-3.

## Background

Epigenetic modifications are recognized for their prominent roles in aging and development [1, 2]. DNA methylation (DNAm), one of the most studied epigenetic marks in humans [3], is strongly associated with gestational age (GA) in newborns and with chronological age in children and adults [4–6]. This property of DNAm has enabled the development of several prediction models, commonly known as ‘epigenetic clocks,’ that are highly predictive of age and GA [6–12]. While it is now firmly established that epigenetic clocks perform exceptionally well in predicting chronological age and, in particular, GA, the reason for the lack of overlap in the selected DNAm sites (CpGs) across different epigenetic clocks has yet to be elucidated.

Current epigenetic clocks are based on variable selection methods such as penalized regression that suffer from two major drawbacks. First, they can be inconsistent in terms of variable selection when the covariates are measured with error and/or noise [13, 14]. Second, if several correlated variables are predictive of the outcome, penalized regression methods tend to select only one among those variables [15]. Given that DNAm is measured with noise [16, 17] and DNAm levels of neighboring CpGs often exhibit correlation [18, 19], the drawbacks of penalized regression methods may likely explain some of the inconsistency observed in the CpGs that are selected by different epigenetic clocks. To overcome these problems, we applied a statistical method called ‘stability selection’ [20] to identify CpGs that are repeatedly selected when predicting GA. In essence, stability selection combines subsampling with a chosen variable selection method, such as the ‘least absolute shrinkage and selection operator’ (lasso), to minimize the number of false discoveries in the set of selected variables.

Epigenetic clocks for GA have tremendous potential for epidemiological and clinical research as accurate predictors of GA and useful surrogates for assessing developmental maturity [21]. However, current GA clocks comprise anywhere between a few dozen to several hundreds of CpGs [7–9, 12], which limit their utility. With current technology, quantifying such a large number of CpGs is too costly and not amenable to most clinical settings. One step towards broader applicability is to construct a more concise and cost-efficient epigenetic clock for GA using as few CpGs as possible without compromising too much on predictive performance. Specifically, this entails selecting the most biologically relevant CpGs while excluding those that mostly capture noise.

Our main aim here was to use stability selection to identify CpGs that are most likely to be stably predictive of GA across samples in an attempt to answer the following questions: i) Are there any CpGs that are stably predictive of GA, and, if yes, do these feature among those in existing GA clocks?; ii) Can the stably selected CpGs be used to build a GA clock consisting of fewer CpGs but that still shows a good performance compared to previously published GA clocks?; and iii) Can we obtain a biologically meaningful interpretation of how the predictive CpGs are linked to GA?

## Results

### Study sample characteristics

The current analyses are based on DNAm data from 2138 newborns from two random subsamples (*n* = 956 and *n* = 1182) within the larger Norwegian Mother, Father, and Child Cohort (MoBa) study [22]. DNAm data in both datasets were generated using the Illumina Infinium MethylationEPIC BeadChip (EPIC). The distributions of GA and sex were similar in the two datasets. GA ranged from 216 to 300 days (mean 279.8 days, SD 11.2 days) in the combined dataset (Table1).

**Table 1:** Characteristics of datasets used for selecting CpGs stably predictive of gestational age

Characteristic	Dataset 1 *n* = 956	Dataset 2 *n* = 1182	Combined *n* = 2138
*GA in days*			
Mean (SD)	279.9 (10.8)	279.7 (11.6)	279.8 (11.2)
Median	281	282	281
Range	216–300	228–300	216–300
Sex (male), n (%)	470 (49%)	569 (48%)	1039 (49%)

*GA* gestational age, *SD* standard deviation

### Twenty-four CpGs were stably predictive of GA

To identify CpGs that are stably predictive of GA, we combined the stability selection methodology proposed by Meinshausen and Bühlmann [20] with lasso regression [23]. We randomly selected 50% of the samples in our combined dataset and performed lasso regression on this subset. This process was repeated 1000 times. We then computed a selection probability for each CpG based on how many times it was selected as being predictive of GA. Finally, the formula derived by Meinshausen and Bühlmann [20] was used to choose a selection probability threshold above which CpGs were defined as being stably predictive of GA. The selection probability threshold depends on the maximum number of false discoveries we could allow on average in our set of stably selected CpGs. A more detailed explanation of the analytic pipeline is provided in the Methods section.

**Fig. 1:**
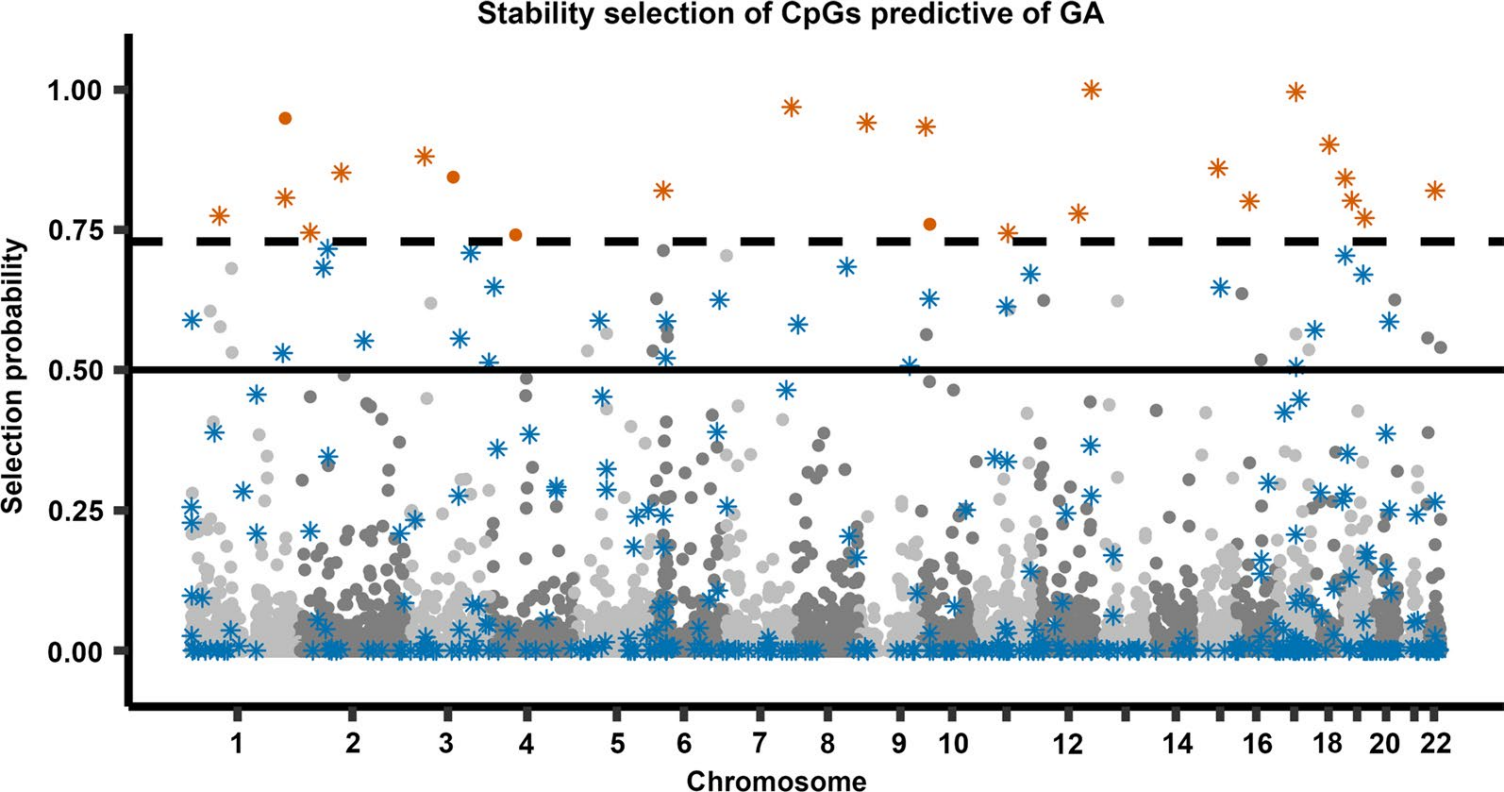
Selection probability of each CpG for the prediction of GA in cord-blood DNAm samples of newborns in MoBa (*n* = 2138). Each point represents a single CpG (*n* = 769,139). The *x*-axis displays the CpGs according to their genomic coordinate, while the *y*-axis represents the selection probability calculated from the stability selection analysis. The solid horizontal line denotes a selection probability of 0.5, where a given CpG has an equal probability of being selected or excluded. The dashed black line denotes the selection probability threshold of 0.73. Asterisks signify CpGs that were selected in previously published GA clocks (specifically, the Haftorn clock [9], the Bohlin clock [7], or the Knight clock [8]). Orange signifies a CpG with a selection probability above the threshold of 0.73, and blue signifies a CpG from a previously published clock with a selection probability below that threshold

Figure 1 shows the 769,139 CpGs included in the analysis and their corresponding selection probabilities. When allowing for a maximum of two false discoveries, which corresponds to a selection probability of 0.73 and above (Additional file 1: Table S1), 24 CpGs were identified as stably predictive of GA (Table 2). The complete output of the stability selection analyses is provided in Additional file 2: Data S1.

**Table 2:** CpGs identified as being stably predictive of gestational age

CpG ID	Selection probability	Chr**	Genomic coordinates**	Relation to CpG Island**	Present on 450 K**	Gene ID**
cg04347477	1.000	12	125,002,007	Island	Yes	*NCOR2*
cg18183624	0.996	17	47,076,904	S_Shore	Yes	*IGF2BP1*
cg25975961	0.969	7	150,600,818	Open sea	No	*–*
cg20320200	0.949	1	217,030,433	Open sea	Yes	*ESRRG*
cg11387576	0.941	9	18,260,848	Open sea	No	*–*
cg11579708	0.934	10	13,142,679	S_Shore	No	*CCDC3; OPTN*
cg21180953	0.902	18	42,489,607	Open sea	No	*SETBP1*
cg09709426	0.881	3	45,911,521	Open sea	No	*LZTFL1*
cg07533333	0.860	15	59,793,834	Open sea	No	*FAM81A*
cg07749613	0.852	2	97,073,539	Open sea	Yes	*–*
cg15393909	0.844	3	111,852,242	Open sea	No	*GCSAM*
cg10714639	0.842	19	1,075,104	S_Shore	Yes	*HMHA1*
cg02567958	0.820	22	37,962,818	Island	Yes	*CDC42EP1*
cg12681972	0.820	6	26,225,299	N_Shore	No	*HIST1H3E*
cg01833485	0.807	1	216,860,692	Open sea	Yes	*ESRRG*
cg00840791	0.802	19	16,453,259	Open sea	No	*–*
cg16348385	0.801	16	30,106,822	N_Shore	Yes	*YPEL3*
cg12999267	0.779	12	94,376,970	Open sea	Yes	*–*
cg20301308	0.775	1	65,534,742	S_Shore	Yes	*–*
cg12542255	0.771	19	45,976,195	Island	Yes	*FOSB*
cg20734092	0.760	10	22,546,132	S_Shelf	No	*LOC100130992*
cg12434132	0.745	2	25,268,065	S_Shelf	No	*EFR3B*
cg11436362	0.744	11	67,053,929	S_Shore	Yes	*ADRBK1*
cg03540917	0.741	4	57,686,587	N_Shore	No	*SPINK2*

*Chr* chromosome, *S_Shore* south shore, *N_Shore* north shore, *S_Shelf* south shelf, *450 K* Illumina HumanMethylation450 BeadChip

^**^Information extracted from the Illumina’s Infinium MethylationEPIC v1.0 B4 manifest file. Genomic coordinates are according to the GRCh37 version of the human genome

### Most of the CpGs selected in GA clocks are not stably predictive of GA

**Fig. 2:**
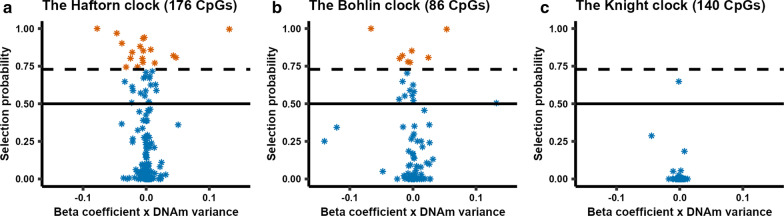
Selection probability of CpGs in our analyses that were selected for being predictive in three previously published GA clocks. **a** The CpGs that were selected in the Haftorn clock (*n* = 176), **b** the CpGs that were selected in the Bohlin clock (*n* = 86), and panel **c** shows the CpGs that were selected in the Knight clock (*n* = 140). In each panel, the *x*-axis displays the beta coefficient for each CpG from the prediction model multiplied by the variance of DNAm in our samples, while the *y*-axis represents the selection probability calculated from the stability selection analysis. The solid horizontal line denotes a selection probability of 0.5 (i.e., a given CpG has an equal probability of being selected or excluded). The dashed black line denotes the selection probability threshold of 0.73. Orange signifies a selection probability above the threshold of 0.73, and blue signifies a clock-CpG with a selection probability below that threshold

To investigate the stability of CpGs selected for GA prediction in previously published GA clocks, we examined three different cord-blood-based epigenetic GA clocks: (i) the ‘Haftorn clock,’ based on EPIC samples [9], (ii) the ‘Bohlin clock,’ based on 450 K samples [7] and (iii) the ‘Knight clock,’ based on 450 K and 27 K samples [8]. In total, 389 unique CpGs in our analyses were previously selected in GA clocks; specifically, 176 in the Haftorn clock, 86 in the Bohlin clock, and 140 in the Knight clock. Of these CpGs, two were in common between the Knight and the Bohlin clock, and 11 were in common between the Bohlin and the Haftorn clock. There were no shared CpGs between the Knight and the Haftorn clock. Eighteen (10.2%) of the Haftorn clock CpGs (Fig. 2a) and eight (9.3%) of the Bohlin clock CpGs (Fig. 2b) were found to be stably predictive of GA. By contrast, none of the Knight clock CpGs were found to be stably predictive of GA (Fig. 2c). Interestingly, four of the CpGs identified as being stably predictive of GA, notably cg03540917, cg15393909, cg20320200 and cg20734092, were not selected by any of the above GA clocks.

### Five CpGs are enough to build a reliable GA clock

**Fig. 3:**
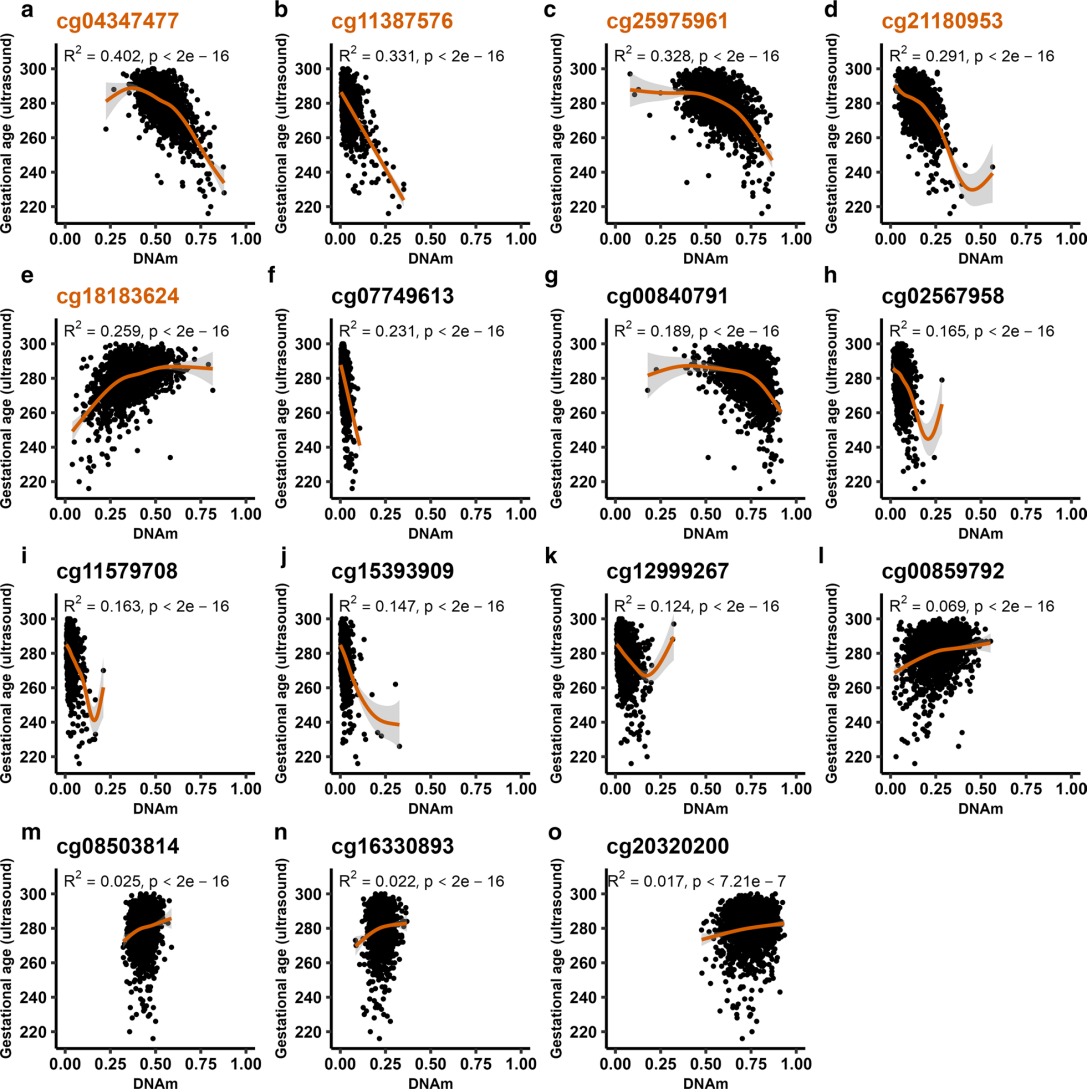
The relationship between DNAm level and GA for each of the 15 stably selected CpGs in the training set (*n* = 1709). In each of the panels (**a**–**o**), ultrasound-estimated GA (*x*-axis) is plotted against the DNAm level (β-value) (*y*-axis) for a given CpG. The orange line indicates the generalized additive model (GAM) regression of DNAm level on ultrasound-estimated GA. Orange CpG titles in panels a-e signify CpGs in the ‘5 stable CpG GA clock’

We investigated whether the CpGs identified as being stably predictive of GA could be used to build an independent epigenetic GA clock based on fewer CpGs but that still shows a similar performance as the previously published GA clocks. We randomly divided the total sample population into a training (80%, *n* = 1709) and test set (20%, *n* = 429), and reran the stability selection analysis on the training set (Additional file 3: Data S2). When allowing for a maximum of two false discoveries, we identified 28 CpGs that were stably predictive of GA in this subset (selection probability threshold = 0.63). To further reduce the number of CpGs, we chose a stricter threshold by allowing a maximum of one false discovery (selection probability threshold = 0.76), which resulted in 15 stably selected CpGs (Fig. 3).

**Fig. 4:**
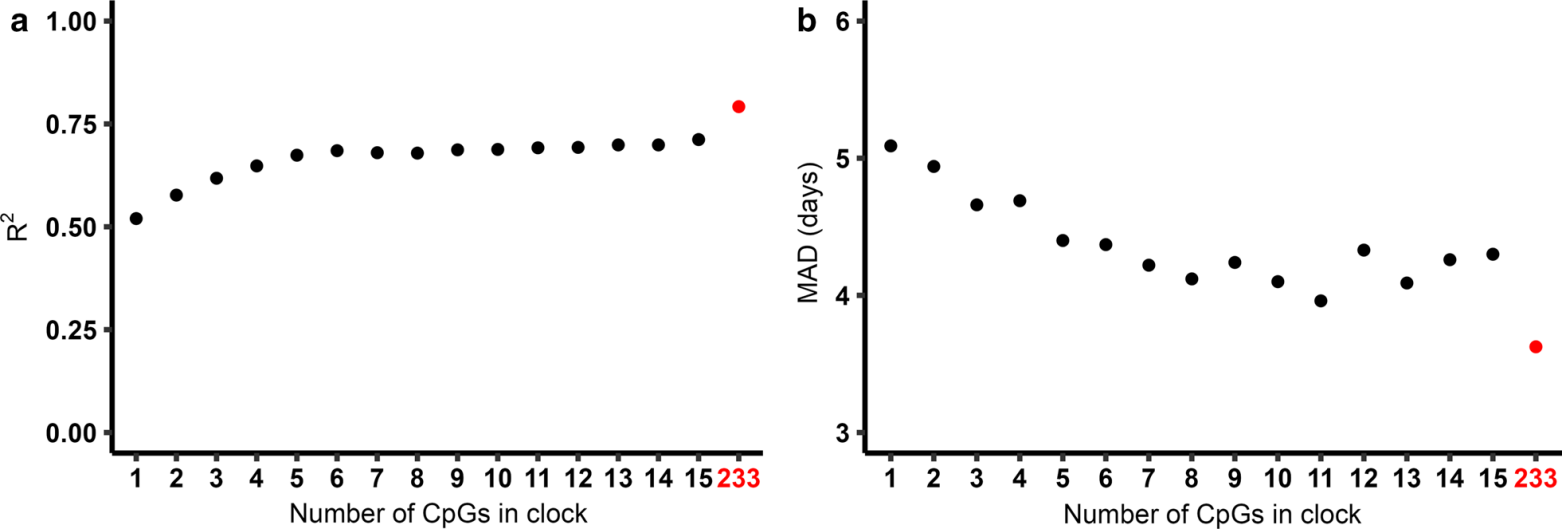
The relationship between the number of CpGs used for prediction and predictive performance in the test set (*n* = 429). Panel **a** shows the R^2^ for each of the clocks and panel **b** shows the corresponding MAD in days. The red dot in each panel shows the predictive performance of a clock developed using the standard framework with lasso

**Fig. 5:**
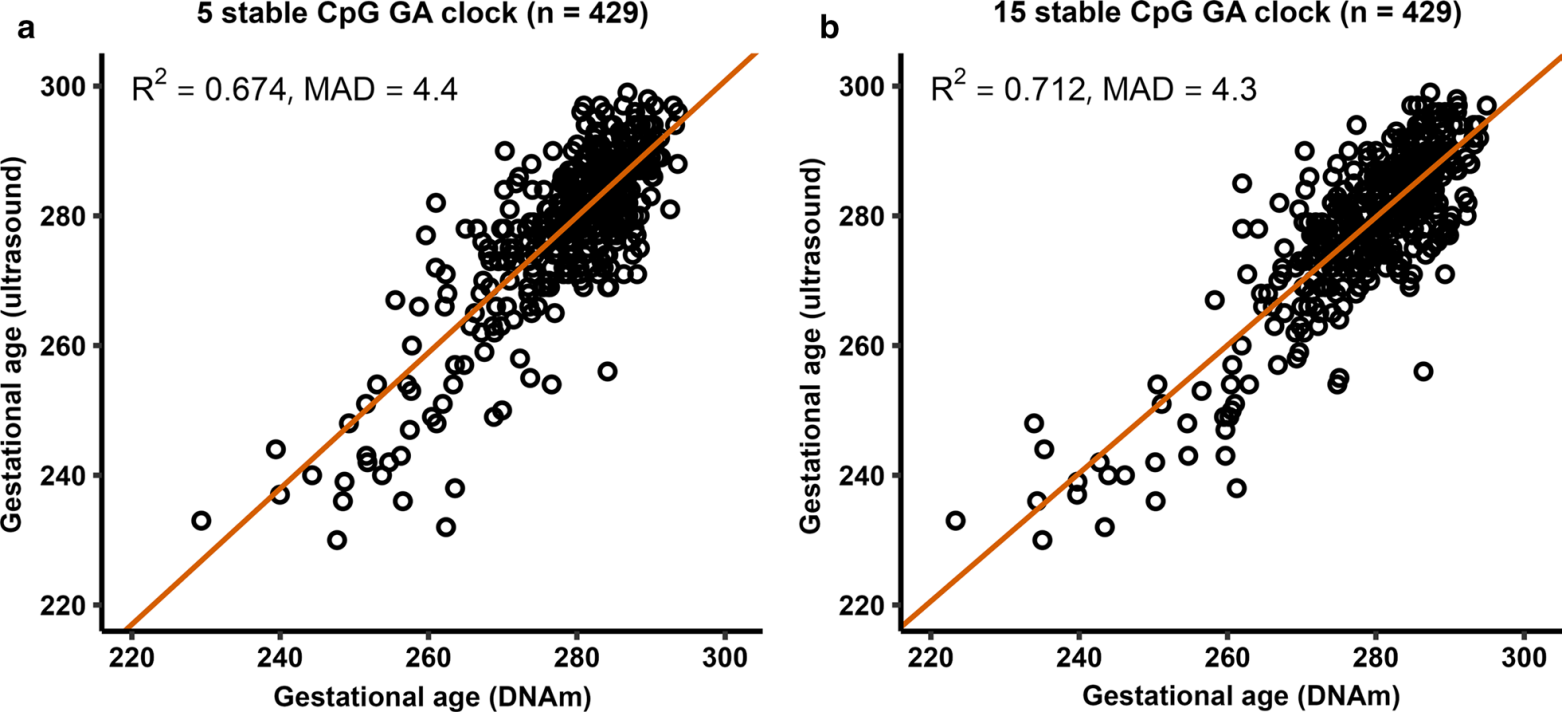
Prediction of GA in the test set (*n* = 429). **a** The scatter plot of GA predicted by DNAm against GA estimated by ultrasound for the ‘5 stable CpG GA clock.’ **b** The corresponding predictions for the ‘15 stable CpG GA clock.’ The orange diagonal line indicates the MM-type robust regression of ultrasound-estimated GA on DNAm-estimated GA

To determine the number of CpGs needed to be included in a GA clock to achieve a similar predictive performance as that of previously published GA clocks, we first fitted generalized additive model (GAM) regressions of GA on DNAm levels in the training set for each of the 15 CpGs identified above and ordered them according to their R^2^ value (Fig. 3). The output of the regression on the CpG with the highest R^2^ was used to predict GA in the test set (*n* = 429). This procedure was iterated by fitting a GAM regression of GA on DNAm levels of the two CpGs with the highest R^2^, then the three CpGs with the highest R^2^, and so on and so forth, until we had constructed 15 different prediction models for GA. We then assessed predictive performance in the test set by comparing R^2^ and median absolute deviation (MAD) for each of the 15 prediction models as well as one that was developed using a standard framework with lasso (Fig. 4; Additional file 1: Table S2). When the predictive performance of the lasso model (with 233 CpGs) was compared to that of the rest of the clocks, it was evident that very few CpGs were needed to attain a sufficiently good prediction of GA. The top CpG (cg04347477) alone predicted GA with an R^2^ of 0.52 and a MAD of 5.09 days. When including five CpGs (cg04347477, cg11387576, cg25975961, cg21180953 and cg18183624) in the ‘5 stable CpG GA clock,’ we obtained an R^2^ of 0.674 and a MAD of 4.4 days. These metrics are comparable to those of the Bohlin clock (*R*^2^ = 0.66, standard error ± 12.5 days (95% prediction interval)) wherein 96 CpGs were needed for prediction [7]. When using all 15 CpGs for prediction, R^2^ increased only slightly, to 0.712 (MAD = 4.3) (Fig. 5), suggesting that the five CpGs in the ‘5 stable CpG GA clock’ explain a remarkably high proportion of the variance in GA. Panels a-e in Fig. 3 depict the relationship between GA and DNAm level of each of these five stably selected CpGs in the training set.

### Some of the predictive CpGs exhibit a nonlinear relationship with GA

**Fig. 6:**
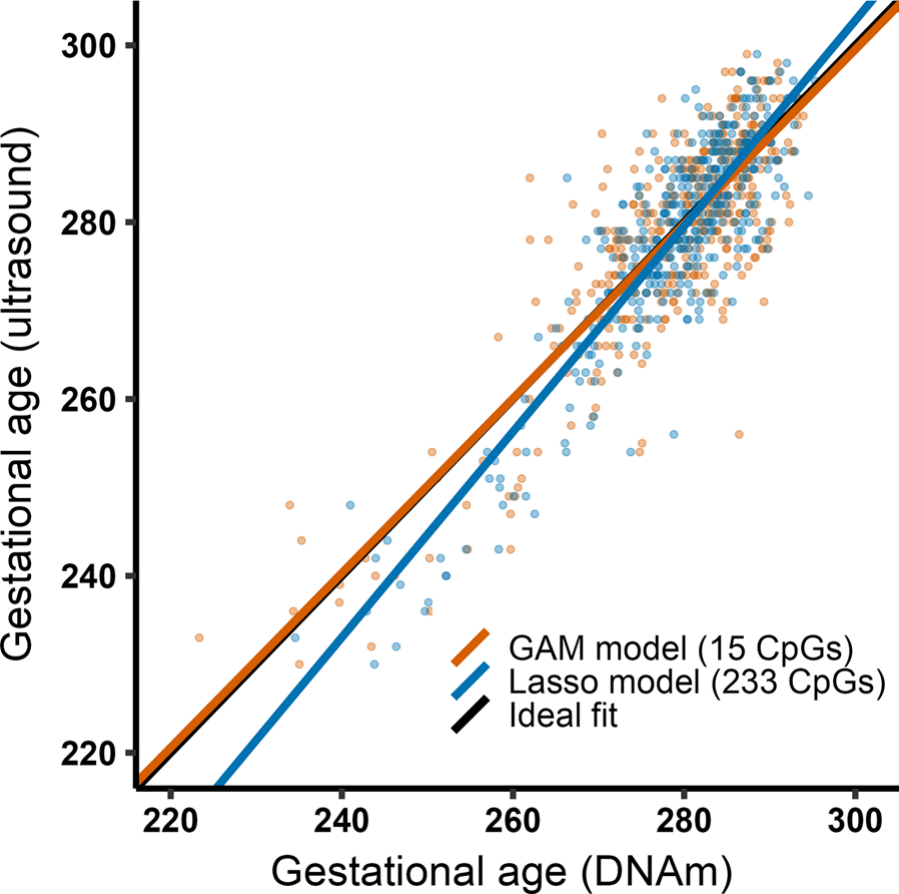
Prediction of GA using a GAM model versus a lasso model. Regression lines showing the relationship between ultrasound-estimated GA and predicted GA in the test set (*n* = 29) using a GAM model including 15 CpGs (orange line) and a lasso model including 233 CpGs (blue line). The black line indicates the ideal fit between ultrasound-estimated GA and DNAm-predicted GA

When building clocks using stably predictive CpGs, GAM was used instead of regular linear regression to account for the observed nonlinearity in the relationship between DNAm and GA. The effective degrees of freedom (EDF) estimated from the GAM were used as a proxy for the degree of nonlinearity in the relationships between DNAm levels and GA [24]. The EDF for the 15 CpGs ranged from 1 to 8.6, with 12 of the CpGs exhibiting an EDF higher than 1, indicating a nonlinear relationship (Additional file 1: Table S3). Only three of the CpGs had an EDF of 1, which is equivalent to a linear relationship. Moreover, the nonlinear relationships between DNAm and GA seem to have a larger effect on the precision of GA prediction in preterm compared to term newborns (Fig. 6).

### Gene and regulatory region annotations of CpGs stably predictive of GA

**Fig. 7:**
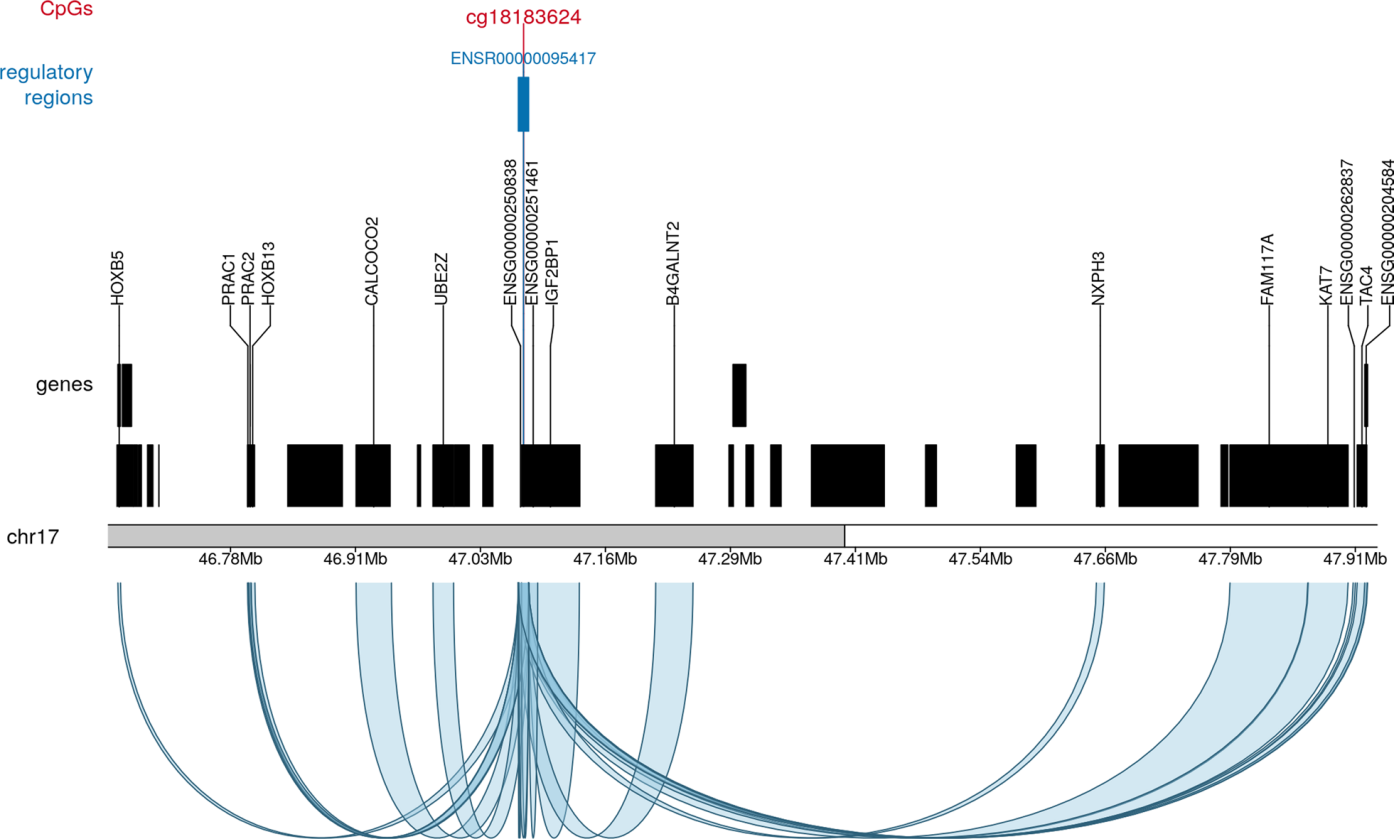
An illustrative example of the regulation map for cg18183624 on chromosome 17. The CpG, shown in red, is encompassed by the regulatory region ENSR00000095417 (blue-colored vertical bar). Below the regulatory region, all the genes are marked as black rectangles and those controlled by ENSR00000095417 are labeled by their gene symbols. The curves underneath the ideogram represent regulatory relationships between ENSR00000095417 and the genes, as predicted by GeneHancer

We searched the *Ensembl* genome browser [25] to check whether the CpGs selected as being stably predictive of GA are located in or near genes or regulatory regions of known pathway annotations. Details on the regulatory region annotation of the remaining stably selected CpGs can be found in Additional file 1: Table S4 and in our GitHub repository. Almost half of the stably selected CpGs are located in promoter regions (*n* = 11, 46%). Table 3 presents a more detailed description of the gene and regulatory region annotations of the CpGs selected for the ‘5 stable CpG GA clock.’ Three of the CpGs in this clock are located in or near specific genes: cg04347477 in *NCOR2*, cg21180953 in *SETBP1 and* cg18183624 in *IGF2BP1*. Moreover, all five CpGs are linked to one or more regulatory regions. cg18183624, for example, is located in a region controlling a small cluster of different genes, several of which are implicated in prenatal development (*IGF2BP1* [26], *KAT7* [27], *HOXB13* and *HOXB5* [28]) immune responses (*TAC4* [29], *CALCOCO2* [30]), in addition to multiple regions encoding long non-coding RNAs (lncRNAs) (*ENSG00000250838*, *ENSG00000262837, NFE2L1-DT, ENSG00000251461*) (see Table 3; Fig. 7).

**Table 3:** Gene and regulatory region annotation of CpGs in the ‘5 stable CpG GA clock’

CpG ID	Gene (Ensembl annotation)	Gene Ensembl ID	Regulatory region type	Regulatory region Ensembl ID	Genes controlled by regulatory region
cg04347477	*NCOR2*	ENSG00000196498	Promoter	ENSR00001046350	–
cg11387576	*–*	–	Enhancer	ENSR00001448127	*SAXO1, PSMC3P1, HSALNG0070247, RF00017-7032, ADAMTSL1, HSALNG0070244*
cg25975961	*–*	–	Promoter flanking region	ENSR00001734862	–
			CTCF binding site	ENSR00000414350	–
cg21180953	*SETBP1*	ENSG00000152217	Promoter flanking region	ENSR00001902774	*Lnc-EPG5-10, 5MWI_A-078, SETBP1, SLC14A2*
cg18183624	*IGF2BP1*	ENSG00000159217	Promoter	ENSR00000095417	*IGF2BP1, ENSG00000250838, ENSG00000262837; UBE2Z; ENSG00000204584, FAM117A; LOC124904116, KAT7, PRAC1, PRAC2, HOXB13, TAC4, CALCOCO2, HOXB5, NXPH3, NFE2L1-DT, ENSG00000251461, ATP5MC1, LOC124904020, B4GALNT2*

Further, we searched for all the 24 stably predictive CpGs in the EWAS catalog [31] and the EWAS atlas [32]. Many of the CpGs were found in previous studies of GA and preterm birth, of aging in early childhood, and of various pregnancy-related phenotypes like gestational diabetes and prenatal smoke exposure. The whole output from this analysis can be found in our GitHub repository.

## Discussion

We found 24 CpGs to be stably predictive of GA after applying a statistical framework that restricts the number of false discoveries in a set of predictive CpGs selected by penalized regression. The results also suggested that most of the CpGs included in previously published epigenetic GA clocks are dispensable. Furthermore, we showed that the stably selected CpGs can be used to construct new GA clocks based on a substantially smaller number of CpGs than previous GA clocks. Importantly, the new GA clocks retained a similar predictive performance to already established GA clocks. These findings underscore the relevance of feature selection, not only in building more efficient epigenetic clocks for GA as here but also for other outcomes and epigenetic clocks.

Epigenome-wide association studies (EWAS) of GA have unraveled thousands of CpGs across the genome that are associated with GA [4, 7, 8, 33, 34]. However, previous studies have shown that most CpGs exhibit a modest effect size [35]. In theory, the presence of many predictive CpGs, where each explains approximately the same amount of variance, is likely to exacerbate the issue of different GA clocks selecting different CpGs. However, our identification of CpGs that were selected up to 100% of the time in different subsamples and that were also highly predictive of GA strongly indicate that only a handful of selected CpGs are needed to explain a remarkably large proportion of the DNAm variance related to GA.

When we compared our stably selected CpGs to those selected by three previously developed GA clocks, namely the Haftorn [9], Bohlin [7] and Knight [8] clocks, only about 10% of CpGs selected in the Bohlin and Haftorn clocks were stably predictive of GA. Moreover, none of the CpGs in the Knight clock were stably predictive of GA. It is important to note that the Bohlin and Haftorn clocks were both developed using samples from the MoBa study, whereas the Knight clock was trained on a combination of datasets from different cohorts. Additionally, the training set used to develop the Knight clock also differs from the Haftorn and Bohlin clocks with respect to several other important parameters, such as the range of GA, the sample size, and type of DNAm array [36]. A particularly interesting observation in our study is that, even though the Haftorn clock was developed using a subset (*n* = 755) of the samples used in the current analyses and was validated in an external replication cohort, 90% of the CpGs in that clock were not considered stably predictive by the current statistical framework. This implies that most of the CpGs selected in epigenetic clocks developed using conventional penalized regression methods are either a selection of many CpGs that have varying degrees of association with GA individually, or that they are simply false positives (i.e., CpGs that are not directly associated with GA but merely tag along other CpGs that are associated with GA [15]). However, it is important to note that, with the stability selection approach, we may fail to detect CpGs that are highly correlated with each other or are part of larger genetic and/or epigenetic networks. Such CpGs may be selected less frequently individually and, therefore, would not be stably selected, although they might still be predictive of GA.

Epigenetic clocks for GA have substantial clinical potential since they can be used for the accurate prediction of GA and as useful surrogates for assessing developmental maturity [21]. One of the main reasons why existing epigenetic GA clocks have had limited clinical utility thus far is the large number of CpGs needed to be assayed to achieve accurate prediction and the costly infrastructure needed to obtain DNA methylation data from cord-blood DNA. The new epigenetic GA clock presented here, based on only five stably selected CpGs, is a significant methodological advance because it affords a similar precision and accuracy as previous GA clocks while substantially curbing the number of CpGs needed to be tested.

Previously published GA clocks tended to overestimate the GA of preterm newborns [7–9]. A similar tendency was also observed in the standard lasso-based clock developed in this study. One possible reason for this overestimation is the typically lower proportion of preterm compared to term newborns in the training sets. However, the Knight clock, which included a larger proportion of preterm newborns in the training set, also tended to overestimate the GA of preterm newborns [8]. A key advantage of the stability selection framework over lasso and elastic net regression is that it separates the *feature selection* step from the *prediction* step. This enables taking nonlinear relationships into account by using methods such as GAM when building the prediction model [24]. When using GAM to build the clock, the GA predictions for preterm newborns were improved compared to the scenario where only the lasso approach was used. Furthermore, for 12 of the 15 CpGs used to develop stable CpG clocks, the calculated EDF indicated a nonlinear relationship between DNAm and GA. These results suggest that at least some of the predictive CpGs exhibit a nonlinear relationship with GA and that this may be important to account for, especially when applying epigenetic GA clocks to preterm newborns.

Several of the stably selected CpGs are in or near genes that have previously been linked to GA. One example is cg04347477 which had a 100% selection probability in our analysis. This CpG alone predicted GA with an R^2^ of 0.52 and a MAD of 5.09 days in our test set. It is located in the promoter region of the nuclear corepressor 2 gene (*NCOR2,* formerly known as *SMRT*). CpGs in this gene have been identified in multiple EWASs of GA as well as in several GA clocks [4, 7, 9, 34, 37, 38]. *NCOR2* encodes a nuclear receptor corepressor that facilitates transcriptional repression by recruiting histone deacetylase complexes (HDACs) and chromatin-remodeling factors [39–41]. The role of *NCOR2* in GA is not clear, but the protein encoded by this gene is essential for a range of biological processes related to mammalian development [42, 43], regulation of inflammation [44, 45], and metabolic homeostasis and aging [46–48].

CpGs linked to the insulin-like growth factor 2 mRNA-binding protein 1 gene (*IGF2BP1)* have also been consistently associated with GA [4, 7, 9, 34, 37, 38]. cg18183624, located within the promoter region of *IGF2BP1*, was assigned a selection probability of 0.996 in our stability selection analyses. IGF2BP1 regulates the translation of specific genes by binding to their mRNAs and contributing to their stability and storage under both normal and stressful conditions [49]. One of the genes regulated by IGF2BP1 is *IGF2*, which is highly expressed in utero and is essential for fetal and placental growth [50]. In addition, IGF2BP1 is pivotal for the switch between fetal to adult hemoglobin, a process that occurs around birth [26, 51, 52].

Two of the CpGs found to be stably predictive of GA in our study, with a selection probability of 0.949 (cg20320200) and 0.807 (cg01833485), are linked to the estrogen-related receptor gamma gene (*ESRRG*). Like *NCOR2* and *IGF2BP1*, CpGs in or near *ESRRG* have also been identified in several other studies of GA [4, 7, 9, 37, 38]. Estrogens are a group of steroid-based sex hormones that are involved in several important developmental and physiological processes, including cartilage proliferation and growth [53], skeletal muscle development and glucose homeostasis [54], and the development of both male and female reproductive tracts [55]. *ESRRG* also plays a critical role in cardiac developmental maturation, particularly in directing and maintaining the metabolic switch from a predominant dependence on carbohydrates during prenatal life to a greater dependence on oxidative metabolism after birth [56, 57].

Furthermore, we recently showed that the association between DNAm and GA is highly cell-type specific and that most of the GA-associated CpGs were restricted to nucleated red blood cells (nRBCs) [38]. However, when we searched for any overlap between the set of stably selected CpGs and the cell-type specific associations between DNAm and GA, most of the stably selected CpGs do not map to any specific cell type (Additional file 1: Table S5). The stably selected CpGs that were also found to be cell-type specific were either in nRBCs, granulocytes, or both, indicating that biological processes in these cell types may be particularly important for the relationship between DNAm and GA.

## Conclusions

In summary, we identified 24 CpGs that were stably predictive of GA using a statistical framework for variable selection that combines subsampling with penalized regression. These CpGs were located in or near genes and regulatory regions that are relevant for immune responses, metabolism and developmental processes, including changes in hemoglobin expression and metabolic processes that occur in the transition from pre- to postnatal life. We showed that most CpGs in existing GA clocks are not stably selected and are not necessary for accurate prediction of GA. Furthermore, the use of GAM regression for GA prediction revealed that some of the predictive CpGs exhibit a nonlinear relationship with GA. Finally, we used the stably selected CpGs to construct a more parsimonious GA clock based on only five CpGs that showed a similar predictive performance as previous GA clocks, creating new opportunities for a more efficient use of DNAm-based GA estimations in research and clinical settings.

## Methods

### Study population

Participants in this study are from the Norwegian Mother, Father, and Child Cohort Study (MoBa), an ongoing population-based pregnancy cohort study conducted by the Norwegian Institute of Public Health (NIPH) [22]. In total, approximately 114,500 children, 95,200 mothers, and 75,200 fathers were recruited from all over Norway from 1999 through 2008. The MoBa mothers consented to participation in 41% of the pregnancies. Extensive details on the MoBa cohort have been provided elsewhere [22, 58].

**Fig. 8:**
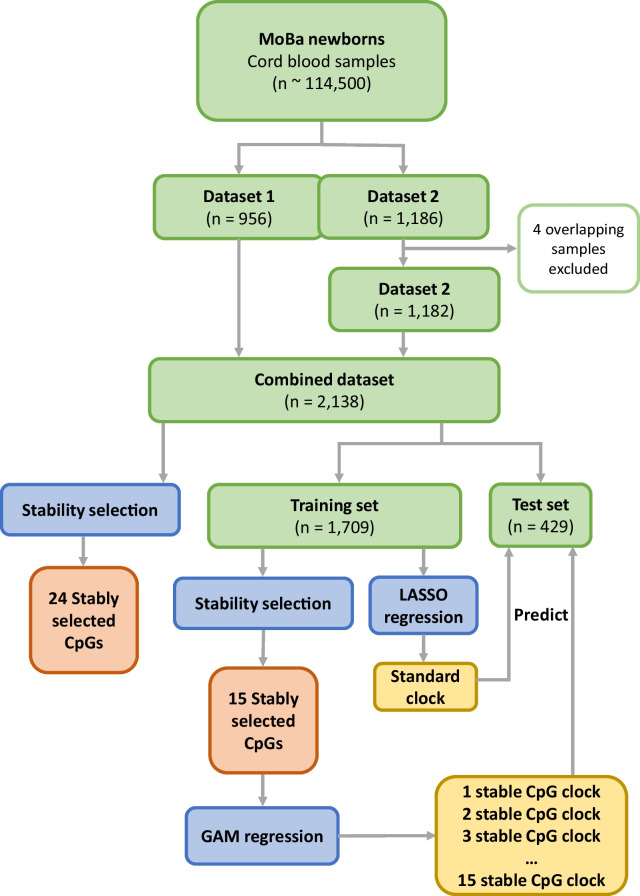
Overview of sample selection and analysis flow. Datasets are highlighted in green, methods in blue, analysis output in orange, and epigenetic clocks in yellow. Two randomly sampled subsets from MoBa (dataset 1 and dataset 2) were included in the current study. Data from four individuals that were present in both datasets were excluded from dataset 2. The two datasets were then merged into a single dataset (‘combined dataset’). The samples from the combined dataset were randomly assigned to a training and test set. Stability selection was performed both on the combined dataset and the training set. Generalized additive model (GAM) regression was used to model the effect of the stably selected CpGs on gestational age (GA) to build clocks based on the stably selected CpGs. In parallel, lasso regression was performed directly on the training set to build a standard GA clock. The standard GA clock and the clocks based on the stably selected CpGs were used to predict GA in the test set

For this study, we used two subsamples of newborns for whom information on ultrasound-estimated GA was available: (i) dataset 1 (*n* = 956) and (ii) dataset 2 (*n* = 1186). Both datasets are based on randomly selected cord-blood samples from the same source population (MoBa). As four individuals were included in both datasets, they were removed from one of the datasets (dataset 2) prior to analysis. The two datasets were then merged into a single dataset comprising a total of 2138 newborns. Figure 8 provides an overview of the sample selection scheme and analysis flow. Detailed characteristics of the study participants and eligibility criteria for dataset 1 have been provided in our recent work [59]. Dataset 2 was sampled in a similar way to make the datasets as compatible as possible.

### DNAm profiling and quality control

Cord-blood samples were taken immediately after birth and kept frozen [58]. The quality control procedures for dataset 1 have been extensively detailed in our previous work [59]. Dataset 2 was processed using the same pipeline to make sure that the two datasets were as compatible as possible. Briefly, DNAm was measured at 885,000 CpG sites using the Illumina Infinium MethylationEPIC BeadChip version 1 (Illumina, San Diego, USA). The raw iDAT files were processed in four batches. Cross-hybridizing probes and probes that had a detection p value greater than 0.01 were excluded. Probes in which the last three bases overlapped with a single-nucleotide polymorphism (SNP) were also removed. BMIQ [60] was used to normalize type I and type II probe chemistries. Samples with low overall signals in control probes were removed after visual inspection, and samples with markedly different DNAm signals than the rest of the samples were also excluded. For consistency, CpG sites excluded from one batch due to poor quality and low detection p value were also removed from all subsequent batches. After quality control, 770,586 CpGs remained in dataset 1 and 795,171 CpGs in dataset 2. 769,139 CpGs were available for analysis in the combined dataset.

### Variables

Information on GA and sex was extracted from the Medical Birth Registry of Norway (MBRN). GA at birth was estimated from ultrasound measurements around week 18 of pregnancy.

### Penalized regression

We used lasso regression from the glmnet R package [61] to select CpGs that are predictive of GA in our samples. Ultrasound-based GA was regressed on the 769,139 CpGs in the combined dataset. Tuning parameter α was set to 1, while λ was selected after tenfold cross-validation.

### Stability selection of CpGs predictive of GA

We combined the stability selection framework proposed by Meinshausen and Bühlmann [20] with lasso regression to identify CpGs that were stably predictive of GA in our total sample of 2138 newborns. By resampling the dataset multiple times, stability selection seeks to identify variables that are repeatedly chosen as predictors while simultaneously controlling the number of selected variables due to noise. We fitted a lasso model (*λ* = 0.386) as described above on a random subsample of n/2 (*n* = 2138) and repeated this process 1000 times. We performed 1000 repetitions, 10 times more than the recommended number [20], because a higher number of repetitions increases the precision of the method. For each CpG, we computed the proportion of runs in which it was selected, which is referred to as the ‘selection probability.’ Finally, we used the following formula (Theorem 1 from Meinshausen and Bühlmann [20]) to choose a threshold that determines the appropriate selection probability threshold for declaring a CpG as stably predictive of GA:

$$E\left( V \right) \le \frac{{q^{2} }}{{(2\pi_{thr} - 1)p}}$$

*E*(*V*) is the expected number of false discoveries in the stably selected set, *q* is the average number of variables (CpGs) selected by the variable selection method (here, lasso), and *p* is the total number of variables included in the analyses (here *n*_CpGs_ = 769,139).

The average number of selected CpGs (*q*) was found by repeating the stability selection procedure with permuted GA values and calculating the average number of CpGs selected (*q* = 593.8). We decided to allow up to two false discoveries on average, resulting in a probability threshold of 0.729. The above approach was repeated on a random subsample of 80% (*n* = 1709) of our original sample of 2138 newborns. This truncated dataset is referred to as the training set. The selected λ for the training set was 0.475 and the chosen probability threshold was 0.764 when allowing up to one false discovery on average (*q* = 450.5).

### Predicting GA from DNAm

The CpGs that were declared stably predictive of GA in the above training set were subsequently used to create prediction models for GA. We used the gam function from the mgcv R package [62] to fit GAM models with GA as the response variable and the stably selected CpGs as the explanatory variables. The effect of each of the CpGs was modeled using a smooth spline.

The output of the GAM regression was used to predict GA in the remaining 20% of our samples—the test set (*n* = 429). Predicted GA was then regressed on ultrasound-estimated GA using MM-type robust linear regression [63] from the R package robustbase [64]. MM-type robust linear regression was used because it is less influenced by outliers than, for example, the ordinary least squares (OLS) regression method [65]. The precision of a given prediction model was defined as the proportion of variance explained by the model (i.e., its *R*^2^ value), while accuracy was defined as the median absolute deviation (MAD, in days) between ultrasound-based and predicted GA.

### Downstream bioinformatics analyses of the selected CpGs

The R package biomaRt [66] was used to fetch annotations for each CpG from the *Ensembl* server (www.ensembl.org) [25], according to the GRCh37 version of the human genome. The *ensembl* regulatory IDs of the regulatory regions identified were then used to manually query the GeneHancer database (https://www.genecards.org/) [67]. The genes predicted to be affected by these regulatory regions were then visually presented using the R package karyoplote R [68]. In addition, we downloaded data from the EWAS catalog [31] and EWAS atlas [32] databases (as of Feb 16th, 2023) and searched for studies involving the stably selected CpGs identified in the current study. We also performed a GOmeth analysis on the 24 stably predictive CpGs. GOmeth is an efficient gene set enrichment analysis method specifically designed for DNA methylation array data [69]. Our set of CpGs was not significantly enriched (FDR < 0.1) in any GO or KEGG categories.

## Supplementary Information

**Additional file 1. Supplementary Table 1**. Relationship between the number of false discoveries, selection probability threshold, and the number of CpGs considered stably predictive of GA;** Supplementary Table 2**. Prediction performance of epigenetic GA clocks developed from stably selected CpGs, in addition to a clock developed using a standard framework with LASSO;** Supplementary Table 3**. Output from a GAM regression of GA on 15 stably selected CpGs;** Supplementary Table 4**. Regulatory region localization of CpGs stably predictive of GA;** Supplementary Table 5**. Overlap between stability selection results and results from a cell-type specific analysis of the association between DNA methylation and GA.

**Additional file 2**. Complete output of the stability selection analyses in the combined dataset.

**Additional file 3.** Complete output of the stability selection analyses in the training set.

## Data Availability

Access to the DNAm datasets can be obtained by applying to the Norwegian Institute of Public Health (NIPH). Restrictions apply regarding the availability of these data, which were originally used under specific approvals for the current study and are therefore not publicly available. Access can only be given after approval by REK under the provision that the applications are consistent with the consent provided. An application form can be found on the NIPH website at https://www.fhi.no/en/studies/moba/. Specific questions regarding access to data in this study can also be directed to Dr. Siri E. Håberg (Siri.Haberg@fhi.no). The data generated in this study are provided in the Supplementary Information.

## Code availability

All statistical analyses were performed using R version 4.1.2 and 4.2.2 [70]. The code used to perform the analyses, as well as R objects containing the stable CpG clocks developed in this study are available on GitHub at github.com/KristineLH/stabsel-clock.
