# Supplementary material for: Stability selection enhances feature selection and enables accurate prediction of gestational age using only five DNA methylation sites
Source: Clin Epigenetics. 2023 Jul 13;15:114. doi: 10.1186/s13148-023-01528-3 (PMC10339624; doi:10.1186/s13148-023-01528-3)
Supplement: Supplementary file 1 — Additional file 1. Supplementary Table 1. Relationship between the number of false discoveries, selection probability threshold, and the number of CpGs considered stably predictive of GA; Supplementary Table 2. Prediction performance of epigenetic GA clocks developed from stably selected CpGs, in addition to a clock developed using a standard framework with LASSO; Supplementary Table 3. Output from a GAM regression of GA on 15 stably selected CpGs; Supplementary Table 4. Regulatory region localization of CpGs stably predictive of GA; Supplementary Table 5. Overlap between stability selection results and results from a cell-type specific analysis of the association between DNA methylation and GA. [file 13148_2023_1528_MOESM1_ESM.pdf]

Supplementary Table 1: Relationship between the number of false discoveries, selection probability threshold, and the number of CpGs considered stably predictive of GA.

E(V)*	Threshold	Number of stably selected CpGs
1	0.9584	3
2	0.7292	24
3	0.6528	34
4	0.6146	44
5	0.5917	47
6	0.5764	53
7	0.5655	55
8	0.5573	59
9	0.5509	62
10	0.5458	62

* The expected number of false discoveries in the stably selected set

Supplementary Table 2: Prediction performance of epigenetic GA clocks developed from stably selected CpGs, in addition to a clock developed using a standard framework with LASSO.

Method	CpGs (n)	R^2	MAD
Stability selection and GAM	1	0.520	5.09
Stability selection and GAM	2	0.577	4.94
Stability selection and GAM	3	0.618	4.66
Stability selection and GAM	4	0.648	4.69
Stability selection and GAM	5	0.674	4.40
Stability selection and GAM	6	0.685	4.37
Stability selection and GAM	7	0.680	4.22
Stability selection and GAM	8	0.679	4.12
Stability selection and GAM	9	0.687	4.24
Stability selection and GAM	10	0.688	4.10
Stability selection and GAM	11	0.692	3.96
Stability selection and GAM	12	0.693	4.33
Stability selection and GAM	13	0.699	4.09
Stability selection and GAM	14	0.699	4.26
Stability selection and GAM	15	0.712	4.30
Standard LASSO	233	0.792	3.63

Abbreviation: MAD, median average deviation

Supplementary Table 3: Output from a GAM regression of GA on 15 stably selected CpGs.

CpG ID	EDF	P-value
cg04347477	2.842	< 2e-16
cg11387576	5.980	0.009
cg25975961	2.675	< 2e-16
cg21180953	7.570	0.001
cg18183624	2.420	8.9e-7
cg07749613	1.000	0.006
cg00840791	2.770	< 2e-16
cg02567958	8.590	< 2e-16
cg11579708	1.000	2.1e-6
cg15393909	1.692	< 2e-16
cg12999267	1.365	0.017
cg00859792	2.322	5.7e-7
cg08503814	2.105	2e-7
cg16348385	1.000	7.8e-5
cg20320200	1.978	2.1e-5

Abbreviation: EDF, effective degrees of freedom

Supplementary Table 4: Regulatory region localization of CpGs stably predictive of GA.

CpG ID	Regulatory region Ensembl ID	Regulatory region type	Genes controlled by regulatory region
cg04347477	ENSR00001046350	Promoter	-
cg18183624	ENSR00000095417	Promoter	*IGF2BP1, ENSG00000250838, ENSG00000262837; UBE2Z; ENSG00000204584, FAM117A; LOC124904116, KAT7, PRAC1, PRAC2, HOXB13, TAC4, CALCOCO2, HOXB5, NXPH3, NFE2L1-DT, ENSG00000251461, ATP5MC1, LOC124904020, B4GALNT2*
cg25975961	ENSR00001734862	Promoter flanking region	-
	ENSR00000414350	CTCF binding site	-
cg20320200	ENSR00001795281	Promoter flanking region	-
cg11387576	ENSR00001448127	Enhancer	*SAXO1, PSMC3P1, HSALNG0070247, RF00017-7032, ADAMTSL1, HSALNG0070244*
cg11579708	ENSR00000024635	Promoter	-
cg21180953	ENSR00001902774	Promoter flanking region	*Lnc-EPG5-10, 5MWI_A-078, SETBP1, SLC14A2*
cg09709426	ENSR00001658177	Enhancer	-
cg07533333	ENSR00001871513	CTCF binding site	-
cg07749613	ENSR00001871514	TF binding site	-
	ENSR00000292361	CTCF binding site	-
cg15393909	ENSR00001973234	Promoter	-
cg10714639	ENSR00001906963	Promoter	-
cg02567958	ENSR00000146024	Promoter	-
cg12681972	ENSR00000195004	Promoter	-
cg01833485	ENSR00001512879	Promoter flanking region	-
cg00840791	-	-	-
cg16348385	ENSR00000085154	Promoter	-
cg12999267	ENSR00000055701	Promoter flanking region	-
	ENSR00001039440	CTCF binding site	-
cg20301308	ENSR00000007928	Promoter	-
cg12542255	ENSR00001912322	Promoter	-
cg20734092	-	-	-
cg12434132	-	-	-
cg11436362	ENSR00001006577	Promoter	-
cg03540917	-	-	-

Abbreviation: CTCF, CCCTC-binding factor

Supplementary Table 5: Overlap between stability selection results and results from a cell-type specific analysis of the association between DNA methylation and GA by Haftorn *et al.* (1).

Stably selected CpG	Selection probability	Gene (Illumina annotation)	Cell type (CpG)*	Cell type (gene)**
cg04347477	1.000	*NCOR2*	*Gran*	*CD4T, gran, NK, nRBC*
cg18183624	0.996	*IGF2BP1*	*Gran*	*Gran, nRBC*
cg25975961	0.969		-	-
cg20320200	0.949	*ESRRG*	-	*nRBC*
cg11387576	0.941		*B-cell, nRBC*	*Bcell, nRBC*
cg11579708	0.934	*CCDC3; OPTN*	-	*nRBC*
cg21180953	0.902	*SETBP1*	*nRBC*	*Gran, nRBC*
cg09709426	0.881	*LZTFL1*	-	-
cg07533333	0.860	*FAM81A*	-	-
cg07749613	0.852		-	-
cg15393909	0.844	*GCSAM*	*Gran, nRBC*	*Gran, nRBC*
cg10714639	0.842	*HMHA1*	-	*nRBC*
cg02567958	0.820	*CDC42EP1*	-	-
cg12681972	0.820	*HIST1H3E*	-	-
cg01833485	0.807	*ESRRG*	-	*nRBC*
cg00840791	0.802		-	-
cg16348385	0.801	*YPEL3*	-	-
cg12999267	0.779		-	-
cg20301308	0.775		-	-
cg12542255	0.771	*FOSB*	-	-
cg20734092	0.760	*LOC100130992*	-	-
cg12434132	0.745	*EFR3B*	-	-
cg11436362	0.744	*ADRBK1*	-	*Gran, nRBC*
cg03540917	0.741	*SPINK2*	-	-

Abbreviations: CD4T, CD4+ T-cell; gran, granulocyte; NK, natural killer cell; nRBC, nucleated red blood cell

* The CpG was significantly associated with GA in this/these cell types(s) in (1).

** Another CpG annotated to the same gene was significantly associated with GA in this/these cell types(s) in (1).

Supplementary References

1. Haftorn KL, Denault WRP, Lee Y, Page CM, Romanowska J, Lyle R, et al. Nucleated red blood cells explain most of the association between DNA methylation and gestational age. Communications Biology. 2023;6(1):224.
